# Histone MacroH2A1: A Chromatin Point of Intersection between Fasting, Senescence and Cellular Regeneration

**DOI:** 10.3390/genes8120367

**Published:** 2017-12-05

**Authors:** Oriana Lo Re, Manlio Vinciguerra

**Affiliations:** 1 Center for Translational Medicine, International Clinical Research Center, St’Anne University Hospital, Brno 656 91, Czech Republic; oriana.lore@fnusa.cz; 2 Faculty of Medicine, Masaryk University, Brno 656 91, Czech Republic; 3 Division of Medicine, Institute for Liver and Digestive Health, University College London (UCL), London WC1E 6BT, UK

**Keywords:** histone variant macroH2A1, fasting, senescence, regeneration

## Abstract

Histone variants confer chromatin unique properties. They have specific genomic distribution, regulated by specific deposition and removal machineries. Histone variants, mostly of canonical histones H2A, H2B and H3, have important roles in early embryonic development, in lineage commitment of stem cells, in the converse process of somatic cell reprogramming to pluripotency and, in some cases, in the modulation of animal aging and life span. MacroH2A1 is a variant of histone H2A, present in two alternatively exon-spliced isoforms macroH2A1.1 and macroH2A1.2, regulating cell plasticity and proliferation, during pluripotency and tumorigenesis. Furthermore, macroH2A1 participates in the formation of senescence-associated heterochromatic foci (SAHF) in senescent cells, and multiple lines of evidence in genetically modified mice suggest that macroH2A1 integrates nutritional cues from the extracellular environment to transcriptional programs. Here, we review current molecular evidence based on next generation sequencing data, cell assays and in vivo models supporting different mechanisms that could mediate the function of macroH2A1 in health span and life span. We will further discuss context-dependent and isoform-specific functions. The aim of this review is to provide guidance to assess histone variant macroH2A1 potential as a therapeutic intervention point.

## 1. Introduction

The histone family of proteins is highly conserved in eukaryotes. DNA is packaged by histones into the nuclei and this is crucial to all DNA-based physical-chemical phenomena. DNA is bound by core histones in the nucleosomes, the functional unit of the chromatin, and there are additional linker histones that bind the DNA in the spaces between nucleosomes. In addition to these classical or “canonical” histones, which make up the bulk of histones in the cell, during evolution, histone variants emerged, with specific properties. With respect to the canonical histones, variants of H2A (H2A.X, H2A.Z.1, H2A.J, H2A.Z.2.1, H2A.Z.2.2, H2A.Bbd, macroH2A1.1, macroH2A1.2 and macroH2A2) and variants of H3 (H3.3, CENP-A, H3.1, H3.2, H3T, H3.5, H3.X and H3.Y) have been isolated in human somatic cells, and two germ line-specific variants of H2B (H2BFWT and TSH2B) have been identified [1]. No variants of H4 have yet been discovered in humans or other higher eukaryotes [1]. Histone variants differ in terms of their gene sequences, as well as the timing and modalities of their processing from RNA to the mature protein, and chromatin deposition during the cell cycle [1]. The reader interested in the evolution of histone variants and their biological role can refer to recent reviews [2,3,4,5,6]. Whereas the coding genes for canonical histones are organized into clusters, histone variants are typically encoded one or two genes only. The unique temporal pattern of expression of each histone variant accounts for its specific cellular functions. A degree of diversity among histone variants is conferred as well by the presence of introns that can be spliced during RNA processing, providing the opportunity to generate alternative splice isoforms and increase transcriptional efficiency. An example of histone variant proteins generated by alternative splicing is macroH2A1. 

**Figure 1:**
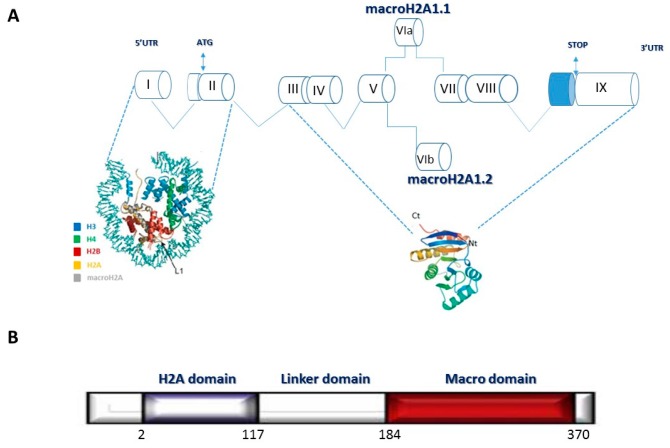
(**A**) Diagram illustrating macroH2A1 gene structure and transcript alternative splicing; (**B**) organization of domains in the protein structure.

MacroH2A1 is composed of a domain 66% homolog to histone H2A, and it stands out because of its unique structure, whereby a *C*-terminal linker connects the histone fold domain to a macrodomain (Figure 1). This domain protrudes from the compact structure of the nucleosome, likely affecting the function and organization of the surrounding chromatin, and is conserved in multiple functionally unrelated proteins throughout the animal kingdom, vertebrates and some invertebrates [7]. MacroH2A1 exists as two alternatively exon-spliced isoforms, macroH2A1.1 and macroH2A1.2 [8] (Figure 1). MacroH2A1.1, but not macroH2A1.2, was originally reported to bind in vitro to *O*-acetyl-ADP-ribose (OAADPR), a small metabolite produced by the protein/histone deacetylase SIRT1 [9]. Historically, macroH2A1 has been implicated in female X chromosome inactivation and transcriptional repression [10,11,12]. MacroH2A1 is enriched and distributed uniformly along the condensed inactive X chromosome, and macroH2A1.2-GFP transgenic ES cells have been generated in order to trace by imaging the process of X chromosome inactivation, allowing the visualization of X chromosome noninvasively in preimplantation embryos and during the mouse life cycle [13].

The view of macroH2A1 as an exclusive chromatin repressor signal has recently been challenged by reports that have linked macroH2A proteins to signal-induced gene activation [14,15,16,17,18]. Moreover, macroH2A1 isoforms have recently taken center stage in the plasticity of stem cell differentiation and in the pathogenesis of many cancers, providing an exciting, yet poorly understood, link to metabolism and nutrients [19,20]. The remainder of this review will focus on discussing the experimental evidence that places histone variant macroH2A1 at the epigenetic crossroad between nutrient sensing, cancer, senescence and cellular regeneration, thus regulating organismal health span.

## 2. MacroH2A1, Cancer and Cellular Senescence

A large amount of data highlighted the primarily tumor suppressive function of macroH2A1, which was reviewed elsewhere [1,19]. Briefly, current data support a tumour-suppressive role for macroH2A1.1, whereas the function of macroH2A1.2 is dependent on the specific cancer context [19]. Artificial over-expression of macroH2A1 reduces the metastatic potential of melanoma and hepatocellular carcinoma (HCC) [14,21,22], whereas small interfering RNA (siRNA)-mediated depletion of macroH2A1 was shown to increase the aggressiveness of HCC, teratoma and breast cancer cells [16,21,23]. This could be due to the fact that absence of macroH2A1 enhances stem like properties in cancer cells, as it is observed in the bladder and in the liver [21,24]. In general, levels of the splice isoform macroH2A1.1 inversely correlate with proliferation. In fact, this variant is downregulated in many cancer types, which is associated with poor prognosis [25,26]. Alternative splicing of macroH2A1 isoforms does not occur in all tumor types; it seems irrelevant in HCC, for instance, where we observed a downregulation of both macroH2A1.1 and macroH2A1.2 at the mRNA and at the protein level [14,21,27,28].

The senescent phenotype was first described more than 50 years ago as a phenomenon characterized by the cessation of cellular division. It is now appreciated that senescence plays an important role in tumorigenesis. Further, senescence is integral to normal biological processes such as embryogenesis and the maintenance of tissue homeostasis. The three major types of cellular senescence, namely, replicative senescence, oncogene-induced senescence and DNA damage-induced senescence (DIS), are distinct because induction of replicative senescence (RS) and oncogene-induced senescence (OIS) were found to be accompanied by aging of primary cells but senescence induced by DNA damage was not, even though RS and OIS activate the cellular DNA damage response pathway [29,30]. These results highlight the independence of cellular senescence from the aging of the cell. In senescent cells, specialized domains of transcriptionally silent senescence-associated heterochromatic foci (SAHF), containing heterochromatin proteins such as HP1, are thought to repress expression of proliferation-promoting genes. Early investigation of the composition and mode of assembly of SAHF and its contribution to cell cycle exit led to the identification of enrichment in macroH2A1 [31]. Chromatin regulators, HIRA and ASF1a drive formation of macroH2A1-containing SAHF and senescence-associated cell cycle exit [31]. 

MacroH2A1 incorporation occurs as a late step and only after SAHF appearance by 4′,6-diamidino-2-phenylindole (DAPI) staining [32]. MacroH2A1 isoforms are thus highly expressed in cells undergoing senescence, which is an antitumor mechanism, suggesting macroH2A1, and in particular macroH2A1.1, may be a useful biomarker for senescent cells in tumors such as lung and colon cancer [26,33]. MacroH2A1 seems to be mechanistically dispensable for RS and DIS, as the number of *β*-galactosidase positive cells in the liver of aged macroH2A1 knock-out (KO) mice is identical to those of aged matched wild type littermates, as well as in H_2_O_2_-treated hepatoma cells KO for macroH2A1 compared to control cells [14]. As stated, cellular senescence is a tumor-suppressive mechanism that permanently arrests cells at risk for malignant transformation. However, accumulating evidence shows that non-dividing senescent cells can have deleterious effects on the tissue microenvironment. The most important of these effects is the acquisition of a senescence-associated secretory phenotype (SASP), which will induce tumor cells or flanking normal cells to become proinflammatory cells. Proinflammatory cells, in turn are capable of further inducing tumorigenesis in neighboring cancer cells [34]. Several types of stimuli can provoke cellular senescence and an SASP. A necessary condition for the occurrence of an SASP is that the irreversible cell-cycle arrest is triggered by severe DNA damage [34]. SASP includes a variety of interleukins (IL-6, IL-15 etc.), chemokines (IL-8, methyl-accepting chemotaxis protein (MCP)s, etc.), growth factors and inflammatory regulators [34]. Two recent studies implicated macroH2A1 in the regulation of the SASP. Chen et al., demonstrated that macroH2A1 act as a tumor suppressor and is a critical component of the positive feedback loop that maintains SASP gene expression, induced by OIS, by triggering the induction of paracrine senescence [35,36]. MacroH2A1 undergoes substantial genome-wide relocalization during OIS (induced by retroviral-mediated expression of H-RasV12), and it is removed from chromatin loci coding SASP genes. The removal of macroH2A1 from SASP genes results from a negative feedback loop involving endoplasmic reticulum (ER) stress, oxidative stress and DNA damage which are involved in the removal of macroH2A1 itself from SASP genes. Thus, macroH2A1 is a critical control point for the regulation of paracrine senescence [36]. In another study, Borghesan et al., found that protein levels of both macroH2A1 isoforms were increased in the senescent livers of very elderly rodents and humans, and in human HCC. MacroH2A1 is thus a biomarker of aging in liver tissue. In response to the chemotherapeutic and DNA-demethylating agent 5-aza-deoxycytidine (5-aza-dC), transgenic expression of macroH2A1 isoforms in HCC cell lines prevented the emergence of a senescent-like phenotype [14]. SASP and whole-transcriptome analyses implicated the p38 MAPK/IL8 pathway in mediating macroH2A1-dependent escape of HCC cells from chemotherapy-induced senescence. In contrast to the study of Chen et al. [36], chromatin immunoprecipitation sequencing uncovered that this escape from senescence in liver cells required activation of transcriptional patterns that were largely independent of the genomic occupancy of macroH2A1 histones [14]. Collectively, these studies suggest macroH2A1 as a novel regulator of oncogene and drug-induced SASP with important implications for disease progression.

A paradigm-making discovery of recent years is that senescence is also a developmental mechanism that contributes to embryonic growth and patterning [37]. Senescence is a normal developmental mechanism found throughout signaling centers in embryonic patterning. Embryonic senescent cells are nonproliferative and share features with OIS, such as SASP factors [37]. Generation of mice models genetically ablated for macroH2A1 did not uncover major developmental phenotypes [38,39]. However, Buschbeck et al., using pluripotent cells and zebrafish models, identified occupancy of macroH2A1 variants at several genes encoding key regulators of development and cell fate decisions, cooperating with Polycomb repressive complex 2, and HOXA cluster genes in a tissue-specific context [1,40]. MacroH2A1 variants, in concert with other histone variants, thus might constitute an important epigenetic mark involved in vertebrate development, and it would be interesting to decipher the reciprocal regulation between macroH2A1 and SASP during normal growth and aging, in absence of oncogenic signals.

## 3. MacroH2A1 and Nutrients

The incorporation of histone variants, and in particular macroH2A1, into the nucleosome is a chief cellular strategy to regulate transcription and cellular metabolism [41]. Energetic homoeostasis and handling of nutrients is a fundamental function of every cell type. In mammals, cellular steatosis is an excessive accumulation of lipids, which reflects an impairment of the normal processes of synthesis and elimination of triacylglycerol fat. Excess lipid accumulates in vesicles that displace the cytoplasm. Although not particularly detrimental in mild cases, large accumulations can disrupt cell constituents and predispose to severe lipotoxicity and evolve to fibrotic states. As the liver is the primary organ of lipid metabolism, it is most often associated with steatosis: (non-alcoholic fatty liver disease (NAFLD); however, it may occur in any organ, particularly the kidneys, pancreas, heart and muscle. Epigenetic changes related to macroH2A variants are involved in human metabolic and carcinogenic diseases (Table 1). We and others reported that macroH2A1 isoforms are found to be upregulated at the protein levels in the liver of mice displaying NAFLD as a simple lipid accumulation or displaying its inflammatory form non-alcoholic steatohepatitis (NASH), induced either by a high-fat diet or by a diet deficient in methionine and choline [28,42]. MacroH2A1.2 is specifically enriched in the liver of NAFLD mouse models, compared with macroH2A1.1 [28]. Both macroH2A1 isoforms are massively upregulated in cryptogenic (i.e., without a known pathogenesis) HCC developing on a steatotic background in humans [28]. Functional studies have shown that macroH2A1.1 and macroH2A1.2 might have different roles in lipid accumulation in human hepatocytes: our biochemical and quantitative imaging analyses showed that ectopic overexpression of macroH2A1.1, but not of macroH2A1.2 is able to protect liver cells against lipid accumulation, both triacylglycerols and cholesterol [43,44].

MacroH2A1.1-overexpressing cells display ameliorated glucose metabolism, reduced expression of lipogenic genes and fatty acid content [43]. These associative studies indicate a possible conserved involvement of macroH2A1 isoforms in lipid metabolism. A number of mechanistic studies using animal have explored this possibility, yielding conflicting outcomes (Table 2). Two mouse models with a macroH2A1 knockout have been reported under a standard diet feeding. In the first model, generated in the pure C57Bl/6J background, developmental changes in macroH2A1-mediated gene regulation were observed [38]: up-regulation of lipogenic genes was detected in the liver of the knockout mice [38], which displayed slight systemic glucose intolerance in the male sex. NAFLD was not observed. This proposed link between lipogenic gene expression in the liver and systemic glucose intolerance is quite surprising since 80–90% of the glucose is taken up by the skeletal muscle, rather than by the liver. The changes in lipogenic gene expression have subsequently been associated with differential physical occupancy of the gene body by macroH2A1 [38,39]. In the second model, knockout of macroH2A1 in a mixed background led to a variable hepatic lipid accumulation in 50% of the females [45]. In this model, the X-linked thyroxine-binding globulin (*TBG*) gene was found to be upregulated in steatotic livers. *TBG* is the main carrier of the thyroid hormone thyroxine (T4), a major regulator of energy metabolism, which could be responsible for the enhanced fat accumulation. Enrichment of macroH2A1 at the *TBG* promoter in female animals indicated that increased *TBG* expression in macroH2A1-knockout mice could be a direct consequence of the absence of this histone [45]. In contrast, our analysis of the in vivo role of macroH2A1 in response to nutritional excess led us to discover that genetic eviction of macroH2A1 confers protection against high fat diet-induced obesity and metabolic derangements in mice [46]. Together, these mice studies did not address the role of the single macroH2A1 isoforms; moreover, whether these histone variants can impact energy turnover in extra-hepatic depots was unknown until recently. In the skeletal muscle, the metabolite-binding macrodomain of macroH2A1 is essential for sustained optimal mitochondrial function but dispensable for regulation of gene expression and cell differentiation [47,48]; the metabolite-binding domain of macroH2A1.1 binds directly poly-ADP ribose polymerase 1 (PARP-1) and inhibits its activity; by doing so it also reduces nuclear NAD^+^ consumption and triggers its overall accumulation [47,48]. The role of macroH2A1 isoforms in adipose tissue plasticity is unknown. In a recent pilot study, we showed evidence that macroH2A1.1 protein levels in the visceral adipose tissue of obese humans positively associate with body mass index (BMI), while macroH2A1.2 is nearly absent [15]. We thus introduced a constitutive green fluorescent protein (GFP)-tagged transgene for macroH2A1.2 in mice, and we characterized their metabolic health upon being fed a standard chow diet or a high fat diet. Despite unchanged food intake, these mice exhibit lower adipose mass and improved glucose metabolism both under a chow and an obesogenic diet [15]. MacroH2A1.2 overexpression in the mouse adipose tissue induced dramatic changes in the transcript levels of key adipogenic genes; genomic analyses comparing pre-adipocytes to mature adipocytes uncovered only minor changes in macroH2A1.2 genomic distribution upon adipogenic differentiation and suggested differential cooperation with transcription factors [15]. MacroH2A1.2 overexpression also markedly inhibited adipogenesis in vitro. Although these data evidenced macroH2A1.2 is an unprecedented chromatin component powerfully promoting metabolic health by modulating anti-adipogenic transcriptional networks in the differentiating adipose tissue, the global picture is puzzling. What is the exact role of macroH2A1 isoforms in fat accumulation and turnover? Since, as described above, depletion of the whole macroH2A1 gene [46] has also, although milder, anti-adipogenic effects in mice fed a HF diet, we argue that macroH2A1.1 has a stronger pro-adipogenic role than the protective one of macroH2A1.2, supported also by the macroH2A1.1-dependent regulation of EZH2/Wnt signaling in differentiating pre-adipocytes [49]. Generation of macroH2A1.1 transgenic murine models will prove its mechanistic role and tissue-specific interaction with macroH2A1.2 during the development of obesity in vivo.

**Figure 2:**
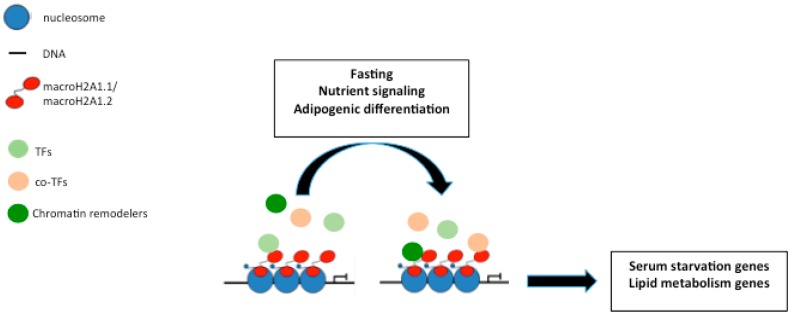
MacroH2A1-dependent integration of nutritional inputs into gene expression programs. This occurs in a fashion that seems independent of changes in its genome occupancy but might require the interaction with yet-to-be identified transcriptional factors (TFs), co-factors and chromatin remodelers (see text for details).

It is thus evident that the expression levels of macroH2A1 isoforms are altered during obesity-related pathologies and that genetic manipulation of these proteins has a strong impact on the development of diet-induced obesity. On the contrary, nutritional strategies based on caloric restriction and fasting have proven to be the only valid strategies to counteract aging-related pathologies and to promote healthy aging. In particular, short-term fasting cycles, or the utilization of fasting mimicking diets, have been shown to diminish cardiovascular and metabolic risk factors in humans, to slow down cancer by empowering chemotherapy (while reducing its side effects) and tissue aging in animal models, as well as to promote cell regeneration and boost of immune systems [50,51,52]. As mentioned above, macroH2A1 can play a positive role in the expression of genes where it is bound in the transcribed region [18]. These studies explored the positive role of macroH2A1 in gene expression and determined that macroH2A1 contributes to the signal-regulated transcription of its target genes [18]. Interestingly, induction of a battery of serum starvation genes in breast cancer cells, normally bound by macroH2A1, is inhibited by knockdown of macroH2A1 [18]. Furthermore, induction of these genes by serum starvation does not lead to changes in macroH2A1 deposition in their bodies, indicating that removal of macroH2A1 is dispensable for transcription triggered by serum starvation [18] (Figure 2). Pehrson’s lab analyzed the macroH2A (macroH2A1 and macroH2A2)-dependent gene regulation in adult liver and the effects of fasting [53]. By using microarrays to examine gene expression in the livers of 2-month-old 129/S6 adult males, upon overnight fasting, the authors show that nutritional status can have a strong effect on macroH2A-dependent regulation of genes involved in nutrient metabolism (for instance: fatty acid binding protein FABP5, *N*-acetyltransferase CML5, etc.) [53]. It appears evident from these studies that macroH2A1 transduces nutritional inputs to gene expression programs in a fashion that seems independent of changes in its genome occupancy but might require the interaction with yet-to-be identified transcriptional co-factors or with chromatin remodelers such as EZH2 [49] (Figure 2). Proteomic and interactome studies are warranted to discover macroH2A1 binding functional partners upon metabolic stress.

## 4. MacroH2A1 and Cell Reprogramming

The function of macroH2A1 in embryonic development and in stem cell biology has been reviewed recently [1]. Here, we will discuss in particular the recent body of evidence indicating a control of somatic cell reprogramming by macroH2A1. Somatic (differentiated) cells can be reprogrammed to an undifferentiated state of pluripotency using different techniques: somatic cell nucleus transfer (SCNT) into an enucleated oocyte or reprogrammed into induced pluripotent stem cells (iPSCs) by a defined subset of transcription factors (OCT4, SOX2, KLF4 and MYC) set up by Yamanaka et al. [54]. The reprogramming process is not efficient and becomes more and more inefficient as cells become differentiated: it is thus believed that the epigenome of somatic cells is stable and it constitutes a barrier to reprogramming. Studies from several groups have now addressed the impact of macroH2A histone variants on the efficiency of reprogramming. During SCNT, macroH2A is rapidly eliminated from the chromosomes of transplanted somatic cell nuclei by a mechanism in which the protein is first physically removed from chromosomes and then degraded; this depletion eventually facilitates reprogramming [55]. A 2012 study from the lab of Sir John Gurdon first demonstrated that macroH2A1 is a marker of cell differentiation during embryogenesis in mice [56]. MacroH2A1 is present at low levels in the inner cell mass and epiblast, but it then gets highly enriched in all differentiated somatic cells further on in mouse development [56]. This study also provided evidence, by chromatin immunoprecipitation (ChIP) experiments, that macroH2A1 physically associates with regulatory regions of pluripotency genes in somatic cells but not in embryonic stem cells; in fact, removing macroH2A1 (or both macroH2A1 and macroH2A2) massively augmented the efficiency of pluripotency [56]. These re-induced pluripotent cells reactivated a pluripotency gene expression program; vice versa, overexpression of macroH2A1 was able to prevent reprogramming of epiblast stem cells to pluripotency [56]. Other studies further explored the link between macroH2A1 epigenetic mark, cell identity and regulation of pluripotency. Two complementary reports from the Izpisua Belmonte’s laboratory using a genome-wide approach showed that macroH2A variants were highly expressed in human somatic cells but downregulated after reprogramming to pluripotency [57,58]. Knockdown of macroH2A histone variants antagonized the in vitro and in vivo differentiation of human iPSC, and this was due to defects in the silencing of pluripotency-related genes OCT4 and SOX2 [58]. At the same time, differentiation genes FOXA2 and SOX17 were not induced [58]. In these reports, ChIP approaches demonstrated that during keratinocytes differentiation, macroH2A1 was recruited to the regulatory regions of pluripotency and developmental genes presenting the H3K27me3 mark, thereby repressing these genes and preserving cell identity [57,58]. Consistently, Bernstein’s laboratory showed that, using fibroblasts derived from macroH2A1/macroH2A2 double knockout mice, macroH2A variants function in a cooperative manner as a barrier that blocks induced pluripotency [59]. MacroH2A1, and also macroH2A2, together with H3K27me3 mark, co-occupy and repressed pluripotency genes in wild-type fibroblasts [59]. This occurred in particular on the downstream genes of UTX, the demethylase that targets K27me3; those genes are induced in the early steps of iPSC reprogramming [59]. Removing macroH2A proteins conferred plasticity: not only did it allow pluripotent cells are able to differentiate properly, but it also allowed differentiated cells to be able to return to a stem-like state [59]. Together, the published studies suggest that macroH2A1 constitutes a “lock” at critical pluripotency genomic sites, and therefore it is an epigenetic barrier that renders cell reprogramming particularly inefficient.

## 5. MacroH2A1 and Integration of Nutritional Signaling into Cell Proliferation and Fate

Genome-wide occupancy studies demonstrated that, in quasi-physiological settings varying from starvation of breast cancer cells [18] to adipocyte differentiation [15], macroH2A1 isoforms do not undergo a significant redistribution on chromatin positioning upon nutritional and differentiation cues (Figure 2). Similarly, in liver cancer cells treated with chemotherapeutic DNA-demethylating agent decitabine, macroH2A1 isoforms are not redistributed on the genome [14]. Conversely, genetic approaches harshly disrupting nuclear lamina resulted in macroH2A1 genomic redistribution [60]; macroH2A1 is a physical linker between repeat sequences and lamin B1, guarding nuclear organization and heterochromatin architecture [61]. Inflammatory stimuli activate nuclear factor κ-light-chain-enhancer of activated B cells (NF-Kb), which in turn has been shown on a nucleosomal template to compete with macroH2A1 binding to the chromatin, although current in vitro and in vivo evidence of this antagonism is weak [21,62]. Moreover, guardians of genome stability involved in cancer such as SWItch/Sucrose non-fermentable (SWI/SNF) helicase, *α*-thalassemia/MR, *X*-linked (ATRX) and aprataxin-PNK-like factor (APLF) act as negative and positive regulators, respectively, of macroH2A1’s chromatin association [63,64].

**Figure 3:**
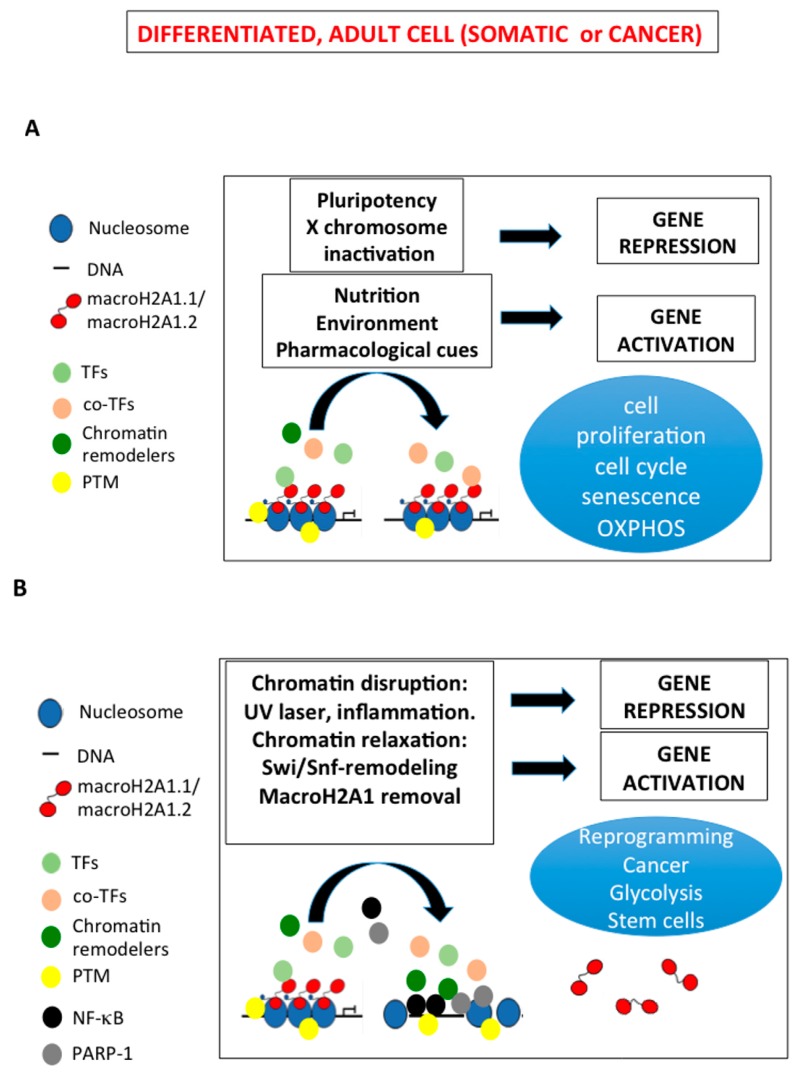
MacroH2A1-dependent integration of nutritional, environmental and pharmacological inputs into gene expression programs and cellular outputs (differentiation, proliferation, cycle, senescence and oxidative phosphorylation metabolism). (**A**) Activity and abundance of macroH2A1 isoforms on specific sites can be modulated by post-translational modification (PTM) and by interactions with neighboring histone PTM, transcription factors, co-TF and chromatin remodelers. (**B**) Disruption of macroH2A1 binding to chromatin by genetic manipulation, ultraviolet (UV) laser-induced localized damage or inflammatory stimuli leads to macroH2A1 removal, resulting in chromatin relaxation, enhanced accessibility to nuclear factor κ-light-chain-enhancer of activated B cells (NF-Kb) and PARP-1-dependent transcriptional activation, and for re-expression of reprogramming genes (see text for details).

Variations in the expression and sequential binding patterns of transcription factors (TF) might mediate potential dynamic interactions with macroH2A1 isoforms on transcriptional templates, as our in silico analysis of adipogenic differentiation program has shown [15]. In addition to interactions between macroH2A1 and TFs [15,65], and with flanking histone marks [18,57,58,59,66], post-translational modification (PTM) is a crucial, and underexplored, mechanism of epigenetic regulation of gene expression by macroH2A1 isoforms in response to nutritional and differentiation cues. A comprehensive steady state mapping of macroH2A1 PTM has been attempted by tandem mass spectrometry and by nanoscale liquid chromatography coupled to tandem mass spectrometry (nanoLC/MS/MS), identifying several ubiquitination, methylation and phosphorylation residues: macroH2A1 could be thus subject to regulation by combinatorial use of covalent modifications as its canonical counterpart H2A [67,68]. Phosphorylation of S137ph residue in the linker region of the protein between the H2A and the macrodomain has been proposed to bind nucleic acids, and to be enriched during mitosis [69]. It is unclear how PTM on macroH2A1 isoforms might affect their function without changes in occupancy. Endogenous macroH2A1 expression is lowly or not expressed in embryonic stem cells, iPSC and cancer stem cells. Based on the experimental data to date, we propose a model in a differentiated and metabolically active adult cell by which macroH2A1 isoforms can regulate distinct subsets of genes, activated upon nutritional, environmental and pharmacological cues and repressed as in the case of pluripotency genes and of X chromosome inactivation, resulting in changes in cell proliferation, cycle, senescence and oxidative phosphorylation metabolism (Figure 3A). Activity and abundance of macroH2A1 isoforms on specific sites can be modulated by PTM and by interactions with neighboring histone PTM and TF (Figure 3A). Disruption of macroH2A1 binding to chromatin by artificial approaches (genetic manipulation), ultraviolet (UV) laser-induced-localized damage or inflammatory stimuli leads to partial/total macroH2A1 removal, resulting in chromatin rearrangement into a more relaxed state, which in turn might be poised for SWI/SNF-dependent chromatin remodeling to enhance accessibility to NF-kB and PARP-1 binding and transcriptional activation, and for re-expression of reprogramming genes, altogether leading to a stem cell phenotype (Figure 3B) [21,57,58,59,61,62,70,71,72,73,74,75,76,77,78]. MacroH2A1 expression and genome occupancy thus lie at a chromatin point of intersection between nutritional status sensing, proliferative and differentiation signals, in turn orchestrating complex transcriptomic responses.

## 6. Perspectives

A consensus of the opinion leaders in the field of histone variants research, highlighted at the 3rd European Molecular Biology Organization (EMBO) Workshop on Histone Variants (6–8 September 2017, Munich, Germany), occurring every 3 years, was that the function of histone variants is highly context-dependent. MacroH2A1, the focus of this review article, makes no exception. MacroH2A1 isoforms mark cellular senescence, and it is unclear if DNA binding activity can be manipulated to revert cellular senescence, which could have anti-aging applications in normal somatic cells. There is a lack of knowledge regarding the differential genomic distribution of endogenous macroH2A1.1 and macroH2A1.2 isoforms, due to the absence of suitable specific ChIP-grade antibodies. A comprehensive picture of the expression patterns of macroH2A1.1/1.2 in different human cell types, normal or cancerous, and at different levels of differentiation, is missing. There is thus great room for further investigation about histone variant deposition (which does not follow any simple rule), metabolism, PTM and in new pathological settings such as in oncogenomics and cancer stem cell biology. Currently, macroH2A1 can be considered a reliable marker for various types of solid cancers, and patents about diagnostic methods for predicting the recurrence of the malignancy [79], as well as for inhibition of DNA-repair enzyme PARP1 [80] have been filed. Advancements in the context-dependent investigation of the basic biological role of macroH2A1, as well as of other histone variants, cannot be disconnected from the development of clinical and therapeutic applications.

## Figures and Tables

**Table 1:** Altered expression of histone variants associated with diseases of lipid homeostasis.

Histone variant	Species	Process/ Disease	Tissue/Cells	Up/Down-Regulation	Reference
MacroH2A	mouse	NASH	liver	Up-regulation	[39]
MacroH2A1.1	mouse	NASH/HCC	liver	Up-regulation	[27]
MacroH2A1.2	mouse	NASH/HCC	liver	Up-regulation	[27]
MacroH2A1.1	human	NAFLD	liver	Up-regulation	[27]
MacroH2A1.2	human	NAFLD/HCC	liver	Up-regulation	[27]
MacroH2A1.2	human	steatosis	HepG2/IHHs	Up-regulation	[41]
MacroH2A1.1	mouse	steatosis	liver	Up-regulation	[41]
MacroH2A1.1	human	obesity	adipose tissue	Up-regulation	[14]
MacroH2A1.2	human	obesity	adipose tissue	Down-regulation	[14]
MacroH2A1.1	mouse	obesity	adipose tissue	Up-regulation	[48]

Non-alcoholic steatohepatitis (NASH); hepatocellular carcinoma (HCC); non-alcoholic fatty liver disease (NAFLD); immortalized human hepatocytes (IHH).

**Table 2:** Effects of manipulating histone variants expression on lipid homeostasis.

Histone variant	Model	Overexpression	Phenotype	Reference
MacroH2A1	mouse	KD	Glucose intolerance, increased hepatic lipidogenic gene expression	[42]
MacroH2A1	mouse	KD	Fatty liver in 50% of females; overexpression of the X-linked thyroxine-binding globuline gene	[44]
MacroH2A1.1	Hepatoma cells	OE	Antilipidogenic	[40]
MacroH2A1.2	Hepatoma cells	OE	Prolidipogenic	[40]
MacroH2A1.1	3T3-L1	OE	Proadipogenic	[14]
MacroH2A1.2	3T3-L1	OE	Antiadipogenic	[14]
MacroH2A1.1	3T3-L1	OE	Proadipogenic	[48]

Knock down (KD); over expression (OE).
